# Activity and Outcomes of a Multidisciplinary Sepsis Unit: Fifty Thousand Consultations over Thirteen Years

**DOI:** 10.3390/antibiotics15080737

**Published:** 2026-07-30

**Authors:** Marcio Borges-Sa, Elena Martin-Martinez, Maria Aranda, Antonia Socias, Sofia Tejada, Alberto del Castillo, Margarita Garau, Luis Pérez de Amezaga, Joana Mena, Andres Giglio

**Affiliations:** 1 Multidisciplinary Sepsis Unit, Son Llatzer Hospital, 07198 Palma, Spain; mborges1967@yahoo.es (M.B.-S.); maranda@hsll.es (M.A.); asocias@hsll.es (A.S.); adelcastillo@hsll.es (A.d.C.); mgarau@hsll.es (M.G.); luis.perezdeamezaga@hsll.es (L.P.d.A.); jmena@hsll.es (J.M.); 2 Health Research Institute of the Balearic Islands (IdISBa), 07120 Palma, Spain; sofia.tejada@idisba.es; 3 Faculty of Medicine, Balearic Islands University, 07122 Palma, Spain; emartinuib@gmail.com; 4 Fundación Codigo Sepsis, 46003 Valencia, Spain; 5 Critical Care Center, Clínica Las Condes Hospital, Santiago 7591046, Chile; 6 Faculty of Medicine, Finis Terrae University, Santiago 7640471, Chile

**Keywords:** sepsis, sepsis code, multidisciplinary sepsis unit, organization of care, in-hospital mortality, early detection

## Abstract

**Background**. Sepsis is a leading, time-dependent cause of in-hospital death, and guidelines now prioritize organized care such as code-sepsis protocols. Yet little is known about the long-term activity, organization and outcomes of permanent, hospital-scale programs. We characterized the activity of a hospital-wide multidisciplinary sepsis unit (MSU) over thirteen years and evaluated mortality trends. **Methods**. Retrospective cohort study using the registry of a hospital-wide MSU (2011–2023). The analysis unit was the sepsis event. Activity, referral pathways, interventions, follow-up and mortality were analyzed; temporal trends used multivariable logistic regression and standardized mortality ratios (SMR). **Results**. The unit attended 10,874 patients across 15,723 events and 50,925 consultations (median age 67; 59.3% men), referred mainly through the sepsis code (45.0%) and, increasingly, proactive early-warning detection (15.2%). An antibiotic change was recommended in 57.2% of events, and 89.8% of all suggested changes were implemented. Follow-up was sustained (median 3 visits; 61.8%). In-hospital mortality was 7.6%, and 11.8% in protocol-confirmed sepsis. Crude mortality did not fall, but severity rose (organ dysfunctions 1.67 to 2.72; septic shock 8 to 13%; both *p* < 0.001) with stable age; after adjustment the calendar-year effect disappeared (adjusted OR 1.00/year, *p* = 0.70) and the SMR stayed near 1.0, including pandemic years. Age, ICU admission (OR 2.65) and organ-dysfunction count (OR 1.37) independently predicted death. **Conclusions**. Over thirteen years, this hospital-wide MSU delivered large-scale, multi-channel, longitudinal care to an increasingly severe population while maintaining stable risk-adjusted mortality, supporting the long-term feasibility of a model integrating early detection, multidisciplinary decision-making, antimicrobial stewardship and follow-up.

## 1. Introduction

Sepsis, defined as life-threatening organ dysfunction caused by a dysregulated host response to infection [1], remains one of the leading causes of death among hospitalized patients worldwide. Despite major advances in critical care and infection management, the burden of sepsis remains substantial. After two decades of decline, the Global Burden of Disease program [2] reported a renewed increase in sepsis incidence and mortality during 2020–2021, with the greatest impact observed among older adults. Even before this resurgence, sepsis was implicated in approximately one in five deaths worldwide [3]. Mortality remains high even in well-resourced healthcare systems, particularly among patients with septic shock [4]. Because sepsis is a time-dependent emergency, clinical outcomes are strongly influenced by how rapidly it is recognized and how effectively coordinated care can be delivered [4].

Improving outcomes in sepsis depends not only on specific therapeutic interventions but also on how healthcare is organized. Early recognition, prompt treatment, and coordinated management have consistently been associated with better clinical outcomes. In healthcare settings where emergency care is based on mandatory protocol-driven pathways, more rapid implementation of sepsis bundles has been associated with lower risk-adjusted mortality [5], and large-scale quality-improvement programs have demonstrated similar benefits across healthcare systems [6]. Reflecting this evidence, the 2026 Surviving Sepsis Campaign guidelines place sepsis performance-improvement programs and code-sepsis protocols among their key recommendations [7]. Consequently, healthcare organizations are increasingly seeking models capable of reliably identifying patients with sepsis and providing guideline-concordant care across different hospital settings and levels of care.

However, most existing sepsis programs focus on specific stages of the patient care pathway. Emergency department sepsis code, ward-based screening systems, and antimicrobial stewardship program each address important, yet relatively isolated components of sepsis care and are often evaluated over limited periods. A multidisciplinary sepsis unit (MSU) represents one such strategy [8]. Typically comprising professionals from intensive care, internal medicine, infectious diseases, microbiology, nursing, and pharmacy, these units aim to facilitate early recognition, coordinate bedside clinical assessment, optimize source control and antimicrobial therapy, and provide longitudinal follow-up throughout the patient’s clinical course rather than limiting intervention to a single episode of care [8,9]. In Spain, multidisciplinary management of severe sepsis has been promoted through national consensus recommendations [10,11,12]. Nevertheless, despite the growing interest in this model of care, detailed descriptions of how these programs function in routine clinical practice, evolve over time, and sustain their activity remain scarce.

Son Llàtzer University Hospital established one of the first hospital-wide MSUs in Spain in 2011 and has prospectively recorded all of its activity in a dedicated electronic registry ever since [9]. Over the years, the program has incorporated several organizational and technological initiatives [13], ranging from nurse-led interventions aimed at reducing blood-culture contamination [14] to the implementation of a hospital-wide machine learning detection system [9,15,16]. The combination of a permanent multidisciplinary structure and a continuously maintained registry provides a unique opportunity to examine the long-term functioning of this care model under real-world clinical conditions.

Nevertheless, its overall functioning remains poorly described in the literature. Previous studies have mainly focused on sepsis-code activations, mortality trends associated with specific organizational interventions, or individual therapeutic practices such as antimicrobial de-escalation [9,17,18]. Much less is known about the overall activity of multidisciplinary sepsis program beyond protocol-confirmed sepsis cases, including the volume and characteristics of patients attended, referral patterns across hospital departments, the type and frequency of interventions performed, the extent of longitudinal follow-up provided, and how clinical outcomes evolve as the programs mature. To address these knowledge gaps, thirteen years (2011–2023) of activity from a hospital-wide MSU since its inception will be analyzed, with the aim of characterizing its case-mix, clinical activity, work and to examine how in-hospital mortality changed over time.

## 2. Materials and Methods

### 2.1. Study Design and Reporting

A retrospective cohort study was performed using the prospectively maintained activity registry of the MSU at Son Llàtzer University Hospital, a tertiary teaching hospital in Palma de Mallorca, Spain. The study included all MSU activity recorded between 2011, when the unit began continuous operation, and 2023. Reporting was conducted in accordance with the Strengthening the Reporting of Observational Studies in Epidemiology (STROBE) guidelines for observational studies [19].

### 2.2. The Multidisciplinary Sepsis Unit

The MSU is a permanent, hospital-wide multidisciplinary team comprising specialists in intensive care medicine, internal medicine, infectious diseases, clinical microbiology, pharmacy and nursing. The unit operates across all adult clinical areas of the hospital, including the emergency department, general wards and the intensive care unit (ICU).

The multidisciplinary sepsis unit brings together several specialties and works as a transversal advisory process: it assesses patients together with the teams in charge of their care, which keep clinical management and prescribing. Clinical care and follow-up are led by internists and intensivists, who follow the full care pathway of each infection. The unit’s care role covers the rapid sepsis response; the follow-up, which spans the infectious and antimicrobial management as well as the resuscitation and management of sepsis; and antimicrobial stewardship, roles that in other health systems are divided among separate teams. Follow-up lasts throughout the whole infectious process, until the infection resolves. Clinical microbiology and pharmacy take part in the unit and in the stewardship program: microbiology triggers referral when cultures are positive and advises on the basis of the results, while pharmacy carries out the pharmacological follow-up of antimicrobials, including dosing and therapeutic drug monitoring. The unit’s nursing staff supports the bedside nursing teams in blood-culture collection, antimicrobial administration and early management, and teaches the nursing teams. Source control is carried out by the treating teams after joint assessment.

Referrals to the MSU may occur through several complementary pathways, including activation of the institutional sepsis code, microbiology alerts (e.g., positive blood cultures), electronic early-warning alerts embedded within the hospital information system, and direct requests from treating physicians or pharmacists [9,13]. Following referral, a bedside clinical assessment is performed (index consultation), and recommendations regarding diagnosis, source control and antimicrobial management are provided. When clinically indicated, patients undergo follow-up assessments during the same episode of care. Follow-up is a serial reassessment, performed in person or through the electronic health record, that monitors clinical and laboratory courses, adjusts antimicrobial therapy, tracks culture results and supports the treating team in the comprehensive management of the patient until antibiotic discontinuation or hospital discharge. Follow-up is therefore longitudinal across the admission rather than a reassessment at a fixed interval.

All consultations, including both index and follow-up assessments, are prospectively recorded in a structured electronic registry. Information related to sepsis-code activations and associated severity indicators, including organ dysfunction and septic shock, is recorded in a linked clinical database (PIMIS), which served as the source of severity-related variables for the present study.

### 2.3. Sepsis Definition

Throughout the study period, the MSU used a consistent operational definition of sepsis based on modified Sepsis-2 criteria, defined as the presence of at least two systemic inflammatory response (SIRS) criteria together with infection-related organ dysfunction. Although Sepsis-3 definitions were introduced during the study period, the registry continued using a stable operational definition to preserve longitudinal comparability across all years [9].

### 2.4. Study Population and Unit of Analysis

The unit of analysis was the sepsis event defined by an index consultation corresponding to the first contact between the MSU and the patient for a given episode. Individual patients could contribute more than one event during the study period. To distinguish new episodes from the ongoing management of the same infectious process, two index consultations in the same patient were considered separate events only when they occurred at least 48 h apart. This threshold was established a priori by the unit to distinguish new episodes from ongoing management of the same infectious process. It was predefined within the registry methodology and applied consistently throughout the study period. Index consultations occurring within less than 48 h of a previous index consultation were considered duplicate registrations and reclassified as follow-up consultations within the same event. The threshold therefore functioned primarily as a de-duplication rule for index consultations that had been entered in place of a follow-up, rather than as a clinical criterion for dividing an ongoing episode.

To improve data consistency, additional quality-control procedures were applied. When an episode contained follow-up consultations but no registered index consultation, a situation considered a recording error, the earliest follow-up consultation was reclassified as the index consultation for that event. Conversely, events without any identifiable consultation record were excluded from analysis.

Eligible events included patients aged 14 years or older with an index consultation recorded between 2011 and 2023. Records from 2010, corresponding to the registry implementation phase and comprising an incomplete year of activity, were excluded. Events involving patients younger than 14 years were also excluded.

Because the study focused on describing the activity and outcomes associated with MSU interventions, analyses were conducted at the event level rather than at the patient level. To account for the possibility that individual patients could contribute more than one event during the study period, the robustness of mortality analyses was subsequently evaluated using models that accounted for within-patient correlation.

### 2.5. Data Sources and Linkage

Data were obtained from three complementary sources. The MSU electronic consultation registry provided information on index and follow-up consultations, including consultation dates, requesting service, referral pathway, infectious focus, sepsis type and recorded clinical interventions. The hospital discharge database provided demographic characteristics, discharge status, ICU admission and discharge service. Severity-related variables were obtained from the sepsis-protocol database (PIMIS), including the number of infection-related organ dysfunctions, the presence of septic shock and the SIRS criteria.

Records from the three databases were linked using the unique hospital episode identifier. Because more than one sepsis-protocol record could be recorded during a single hospital admission, severity was assigned at the event level by matching each sepsis event to the temporally closest protocol record within the corresponding admission. This approach was intended to ensure that severity measurements reflected the clinical status most closely associated with the index consultation.

### 2.6. Variables

Activity-related variables included the requesting service, referral pathway, principal infectious focus, sepsis classification, clinician-reported time spent on the index consultation, number of follow-up consultations per event, and the occurrence of two or more consultations for the same patient on the same calendar day.

When multiple infectious focuses were recorded for the same event, a principal focus was assigned according to main clinical symptoms and signs. The classification of infectious focus was performed to ensure that each event contributed a single primary source of infection to the analyses.

MSU interventions were analyzed at the event level and were considered present when recorded either during the index consultation or at any subsequent follow-up consultation. Recorded interventions included antibiotic modification, spectrum reduction, antimicrobial de-escalation, treatment initiation, treatment discontinuation, escalation in response to clinical deterioration, correction of inadequate empirical therapy, switch to oral therapy and dose adjustment.

Interventions were defined operationally as follows and were not mutually exclusive (an event could carry more than one): antibiotic change, any modification of the antimicrobial regimen; spectrum reduction, narrowing of the antimicrobial spectrum; de-escalation, reduction in the spectrum and/or in the number of antimicrobial agents guided by clinical course and microbiological results; treatment initiation, start of antimicrobial therapy in a patient not previously treated; treatment discontinuation, stopping therapy (for example, when infection was ruled out or treatment completed); escalation, broadening of therapy in response to clinical deterioration or inadequate response; correction of inadequate empirical therapy, change because the empirical regimen did not cover the identified or suspected pathogen; switch to oral therapy, transition from intravenous to oral administration; and dose adjustment, modification of dose or interval (for example, for renal function or therapeutic drug monitoring).

Acceptance of a therapeutic recommendation was classified as documentation of the recommended antibiotic modification during either the same consultation or the subsequent consultation within the corresponding event. Recommendation acceptance was derived from prospectively recorded registry data.

Microbiological variables included documented bacteremia and positive microbiological cultures. These variables were analyzed only for events occurring from 2016 onward, when microbiological data collection became systematic within the registry.

### 2.7. Outcomes

The primary outcome was in-hospital mortality. Because discharge outcomes were recorded at the admission level rather than at the event level, in-hospital mortality was attributed to the terminal sepsis event of each admission in order to avoid double counting when multiple events occurred during the same hospital stay.

ICU admission was also obtained from the hospital discharge database and was analyzed as a marker of care intensity and disease severity. As with mortality, ICU admission was assigned to the terminal event of each admission when multiple events occurred during the same hospitalization.

Severity descriptors were obtained from the linked sepsis-protocol database and included the number of infection-related organ dysfunctions and the presence of septic shock. These variables were used to characterize case severity and for risk adjustment in multivariable analyses.

### 2.8. Statistical Analysis

Continuous variables are summarized as mean with standard deviation (SD) or median with interquartile range (IQR) according to their distribution, and categorical variables as counts and percentages. Denominators are reported for different analyses. Missing data was excluded from analysis and no imputation was performed. Groups defined by infectious focus, care level and activation route were compared with the chi-square or Fisher exact test for categorical variables and the Mann–Whitney U or Kruskal–Wallis test for continuous variables. The evolution of in-hospital mortality across the study period was assessed using annual crude mortality with a test for linear trend and a multivariable logistic regression model with calendar year as a continuous term, adjusted for age, ICU admission, infectious focus and severity; the linearity of the temporal and dose–response effects was examined with restricted cubic splines. Performance over time was additionally expressed as the standardized mortality ratio (SMR; observed versus expected deaths, the latter predicted from the severity-adjusted model), with 95% confidence intervals (CI). Multivariable logistic regression was used to identify factors independently associated with in-hospital mortality; robustness to the inclusion of patients with more than one event was checked with a generalized estimating-equation model clustered by patient. Two-sided *p* values below 0.05 were considered significant. Analyses were performed in Python 3.14.5 (pandas, statsmodels).

## 3. Results

### 3.1. Multidisciplinary Sepsis Unit Activity

**Figure 1:**
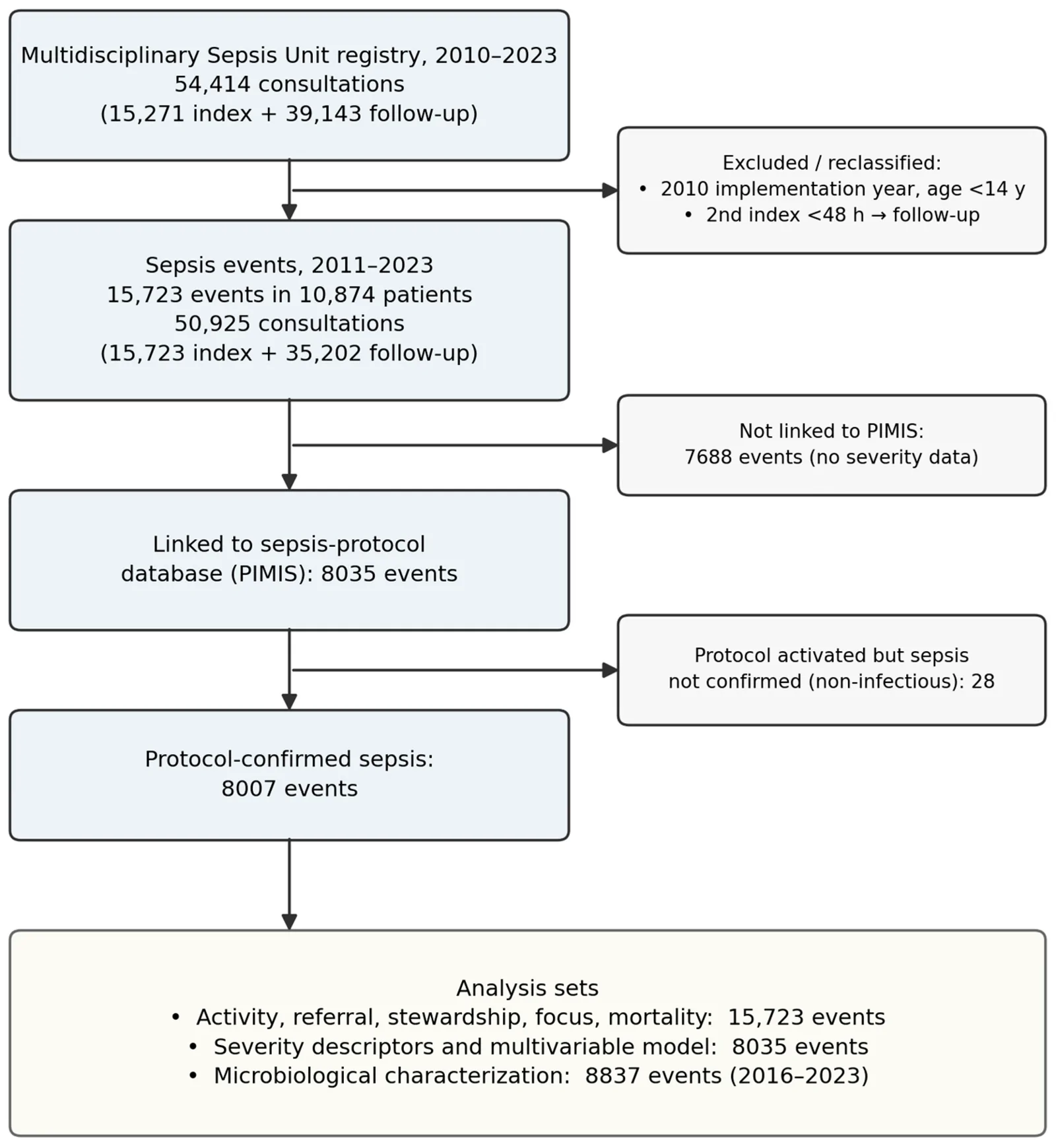
Derivation of the study cohort, from the multidisciplinary sepsis unit registry (2010–2023) to the analysis sets, showing exclusions, linkage to the sepsis-protocol database (PIMIS) and protocol-confirmed sepsis.

The registry contained 54,414 consultations, including those recorded during its initial implementation year (2010). The present analysis was restricted to the period 2011–2023 and to patients aged 14 years or older, yielding 15,723 sepsis events in 10,874 patients and a total of 50,925 consultations, comprising 15,723 index consultations and 35,202 follow-up consultations (Table 1 and Figure 1).

Longitudinal follow-up represented a substantial component of MSU activity. Each event began with an index consultation and was followed by a median of 3 follow-up visits per event (IQR 1–5), while 61.8% of events received at least one follow-up assessment. Overall, follow-up consultations accounted for 69.1% of all recorded contacts.

**Figure 2:**
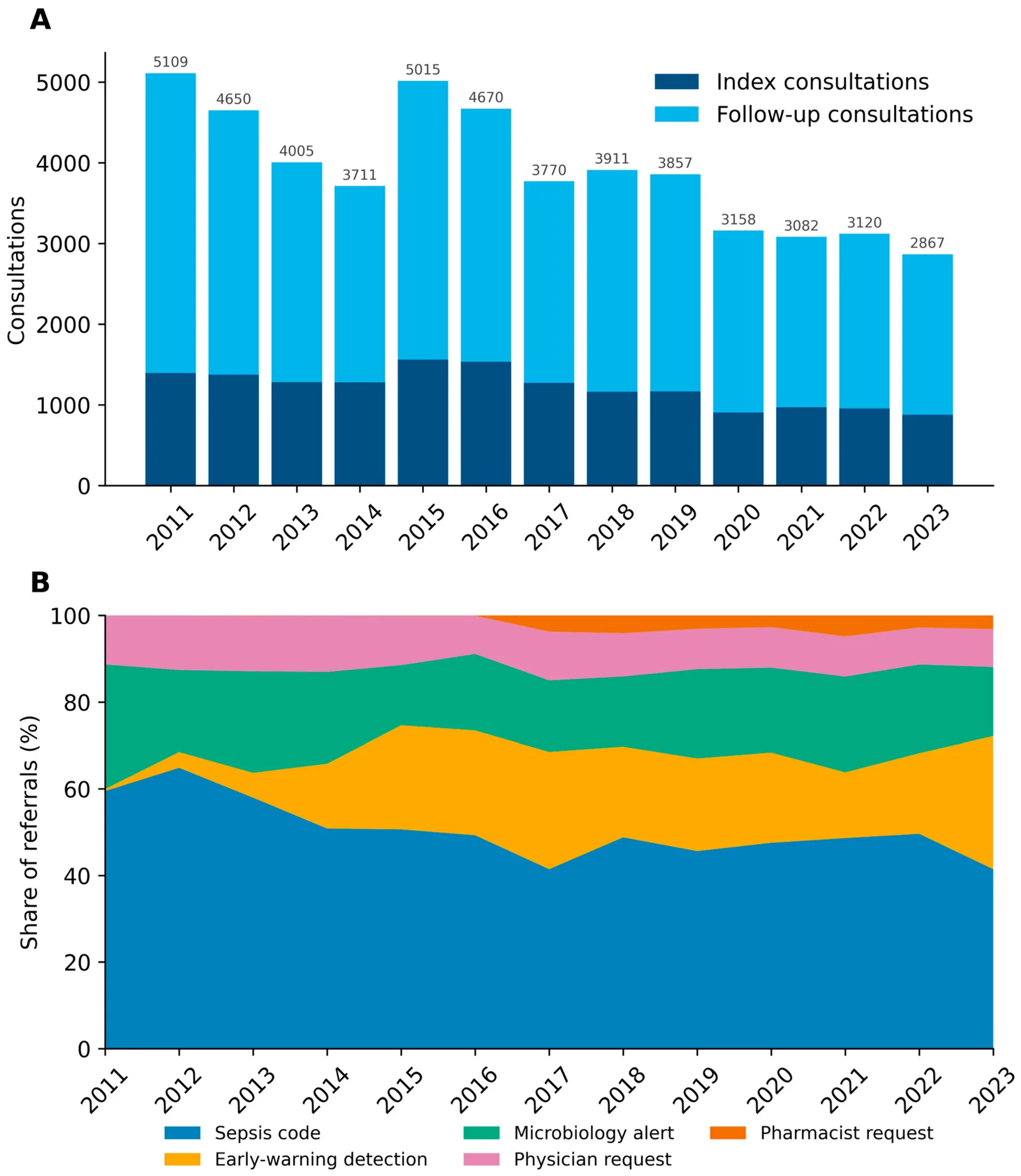
Activity and referral pathways of the multidisciplinary sepsis unit, 2011–2023. (**A**) Annual number of consultations according to consultation type (index and follow-up consultations). Follow-up consultations represented most recorded contacts throughout the study period. (**B**) Relative contribution of referral pathways over time among referrals with a recorded activation route. The contribution of proactive early-warning detection increased progressively over the study period, whereas the relative contribution of sepsis-code activation decreased. Percentages in panel (**B**) are calculated among referrals with a recorded activation route.

Annual activity was highest during the first years of operation, reaching nearly 5000 consultations per year between 2011 and 2015, and subsequently declined to approximately 2900 consultations in 2023 (Figure 2A). Both consultation types declined over the period: index consultations fell from 1395 in 2011 to 878 in 2023, and follow-up consultations from 3621 to 2081. Because follow-up is the larger component, it accounted for most of the absolute reduction. The ratio of follow-up to index consultations nonetheless remained within a narrow range (approximately 2.0 to 2.6) with no clear temporal trend, indicating that the composition of activity was preserved as overall volume fell. Two or more consultations for the same patient on the same calendar day were recorded in 3.5% of events.

### 3.2. Patients and Referral Sources

The median age of the cohort was 67 years (IQR 55–77) and 59.3% of patients were male (Table 1). Referrals originated from a broad range of hospital services. The ICU accounted for 22.2% of consultations, followed by the emergency department (18.3%), surgical services (16.7%), oncology (13.0%) and gastroenterology (7.3%), with the remaining referrals distributed across medical and surgical departments.

Respiratory (27.6%) and abdominal (26.4%) infections were the most frequent infectious focus, together accounting for more than half of all events, followed by urinary infections (15.6%). Catheter-related or primary bloodstream infections, skin and soft-tissue infections, and other sources accounted for smaller proportions of cases. Among events linked to a sepsis-protocol record (n = 8035), the median number of infection-related organ dysfunctions was 2 and septic shock was present in 10.6%. Of the 8035 events linked to a sepsis-protocol record, 8007 were confirmed as sepsis by the unit; the remaining 28 were protocol activations subsequently judged non-infectious (Figure 1).

### 3.3. Detection Channels

Multiple referral pathways contributed to case identification throughout the study period (Table 2). Overall, referrals originated from the institutional sepsis code in 45.0% of events, microbiology alerts in 17.1%, proactive early-warning detection in 15.2%, direct physician requests in 9.4% and pharmacist requests in 1.4%; a further 0.8% came from other sources and 11.0% had no activation route recorded (percentages of all 15,723 events).

The relative contribution of these pathways evolved over time (Figure 2B). The proportion of referrals generated through sepsis-code activation decreased from 55.8% during 2011–2015 to approximately 46% in subsequent years, whereas proactive early-warning detection increased from 10.2% to approximately 21–23% of referrals. Pharmacist-initiated consultations, absent during the initial years of the program, accounted for 3.4% of referrals by the end of the study period. This followed an operational change that incorporated clinical pharmacists into the unit’s case-detection and referral circuit, allowing them to identify candidate patients during stewardship review and to refer them directly. In contrast, microbiology alerts and physician requests remained relatively stable over time.

### 3.4. Clinical Interventions

Microbiological fields were systematically recorded from 2016 onward (8837 events). Within this subset, bacteraemia was documented in 13.1% of events and a positive microbiological culture in 35.5%. In events without a clinically identified focus, documented bacteraemia was less frequent (9.1%) than in the cohort overall.

Antimicrobial stewardship constituted a major component of MSU activity (Table 2). At least one therapeutic recommendation was issued in 9240 events (58.8% of 15,723) and a change in antimicrobial therapy was recommended in 8995 events (57.2%). Across the study period the unit made 15,950 therapeutic recommendations, comprising 16,758 individual suggestions (a single recommendation could contain more than one suggested change).

The most frequent intervention involved optimization of antimicrobial treatment. Spectrum reduction was recommended in 26.1% of events, treatment discontinuation in 15.6%, switch to oral therapy in 14.7%, antimicrobial de-escalation in 13.3%, escalation in response to clinical deterioration in 12.1%, correction of inadequate empirical therapy in 10.3%, and treatment initiation in 7.1% of events (Table 2).

Implementation of MSU recommendations was high. At the level of individual suggestions, 15,045 of 16,758 suggested changes (89.8%) were documented as implemented in the same or the following consultation of the event.

The median clinician-reported time spent on the index consultation was 40 min (IQR 20–60), indicating a substantial level of clinical involvement in the assessment and management of referred patients.

### 3.5. Outcomes

**Figure 3:**
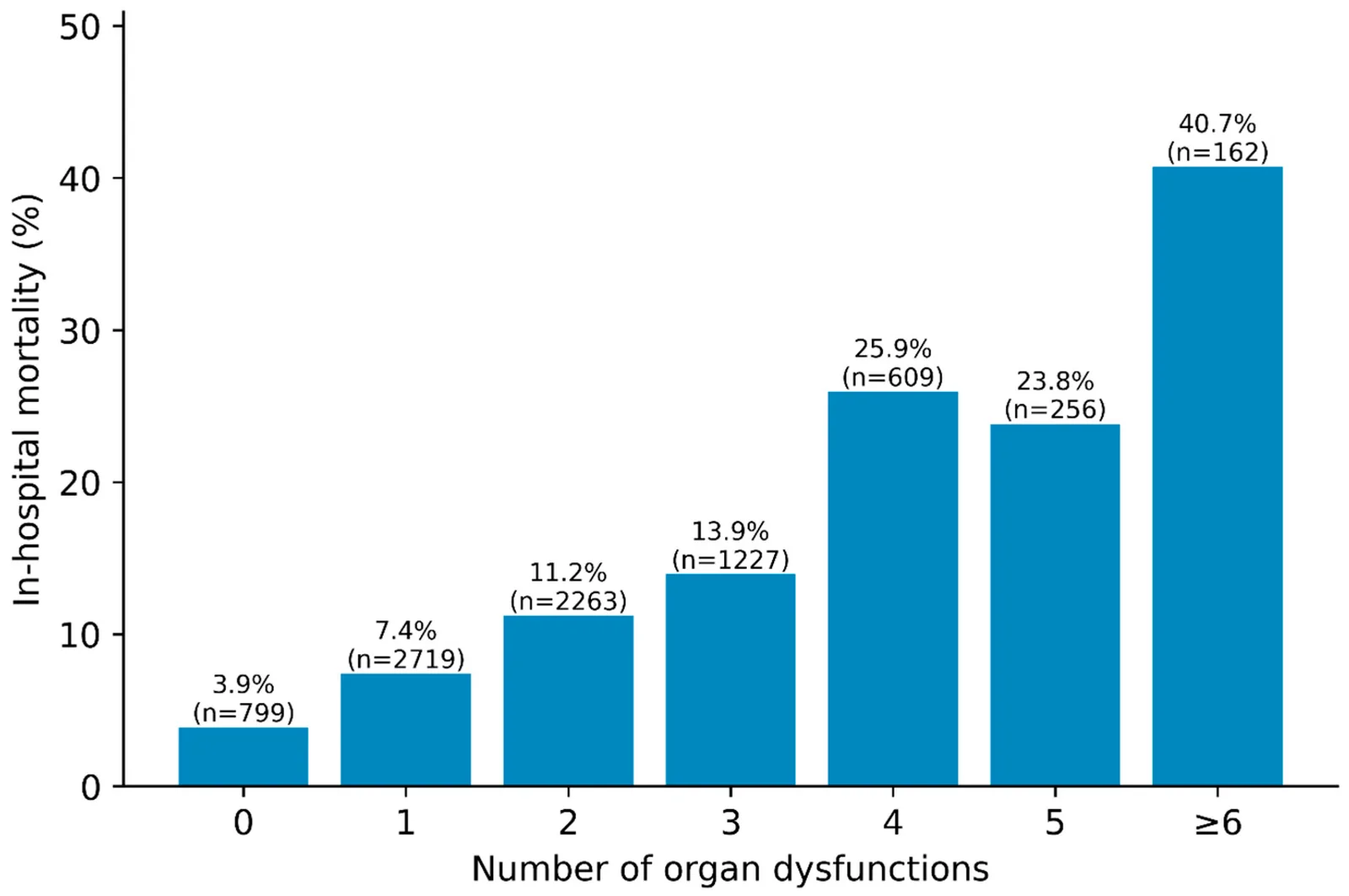
In-hospital mortality according to the number of infection-related organ dysfunctions. Bars represent in-hospital mortality (%) stratified by the number of infection-related organ dysfunctions. Values above each bar indicate mortality (%) and the corresponding number of events (n).

Overall in-hospital mortality was 7.6% across all events (1189 deaths) and 11.8% among protocol-confirmed sepsis events. Mortality increased progressively with severity, rising from 2.2% among events without organ dysfunction to 11.9% among those with six or more organ dysfunctions (Figure 3).

Crude in-hospital mortality varied by infectious focus (Table 3), from 3.8% for urinary infections to 12.0% for events without an identified focus and 10.6% for respiratory infections; a small endovascular/endocarditis subgroup had the highest mortality (23.7%). Urinary infection, the focus with the lowest mortality and a homogeneous clinical presentation, was used as the reference category in the multivariable model. Patients admitted to the intensive care unit experienced higher mortality than those managed outside it (17.4% versus 3.8%).

**Figure 4:**
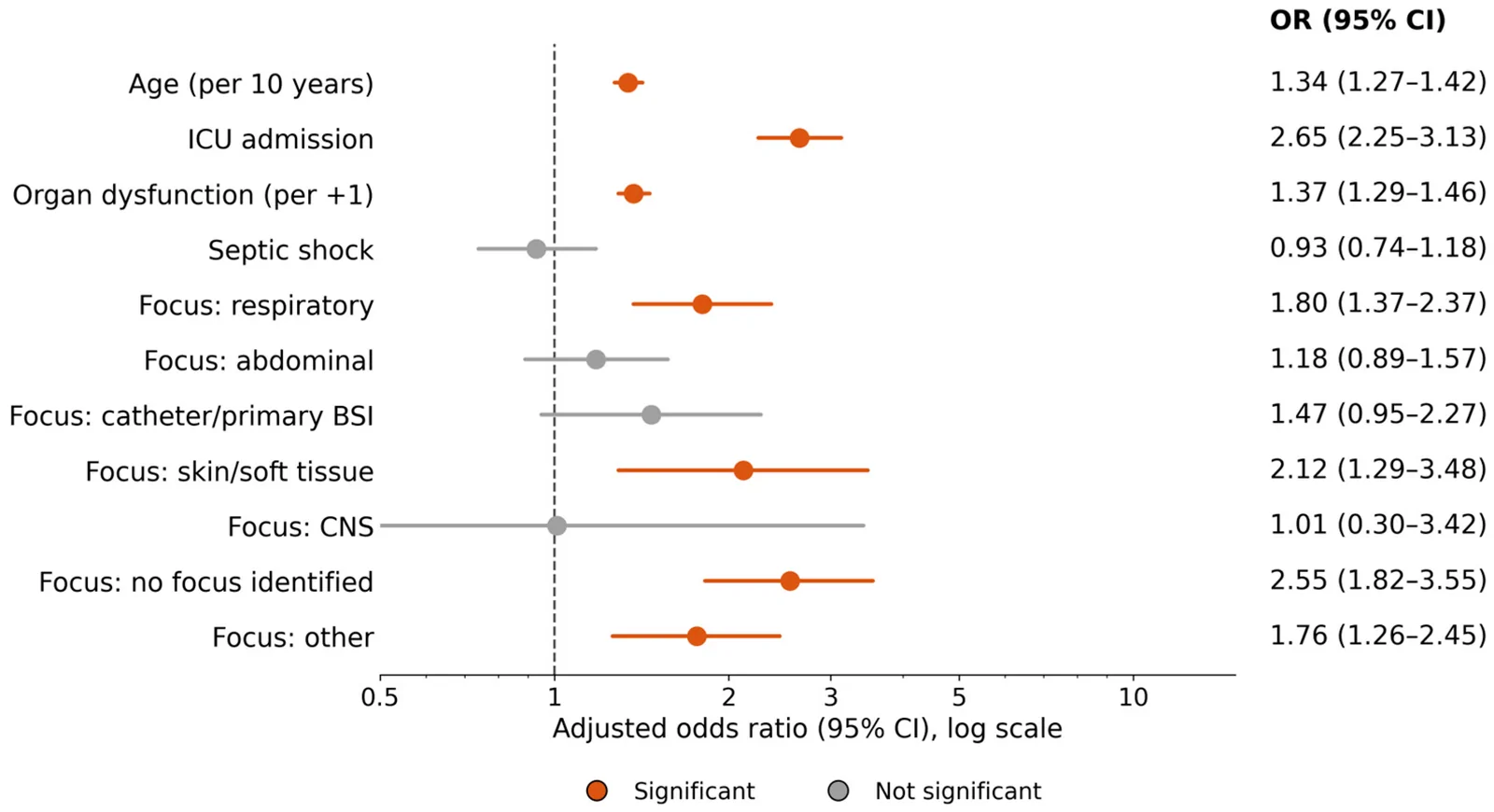
Independent predictors of in-hospital mortality in multivariable logistic regression analysis. Adjusted odds ratios (OR) and 95% confidence intervals (CI) for factors associated with in-hospital mortality among events linked to a sepsis-protocol record (n = 8035). Points represent adjusted ORs and horizontal bars indicate 95% confidence intervals. Orange markers denote statistically significant associations (95% CI excluding 1) and gray markers denote non-significant associations (95% CI including 1) Values to the right of the vertical reference line (OR = 1) indicate increased odds of death. Urinary infection was used as the reference category for infectious focus.

Multivariable analysis was performed in the subset of events linked to a sepsis-protocol record (n = 8035) (Table 4, Figure 4). Age (OR 1.34 per 10 years, 95% CI 1.27–1.42), ICU admission (OR 2.65, 95% CI 2.25–3.13) and each additional organ dysfunction (OR 1.37, 95% CI 1.29–1.46) were independently associated with in-hospital mortality. Compared with urinary infections, respiratory, skin and soft-tissue, and unidentified-focus infections were associated with approximately two-fold higher odds of death. Septic shock was not independently associated with mortality after adjustment for organ dysfunction burden (OR 0.93, 95% CI 0.74–1.18).

The multivariable model showed acceptable discrimination (C-statistic 0.75). Similar estimates were obtained in a generalized estimating-equation analysis accounting for repeated events within individual patients, indicating that the findings were robust to within-patient clustering (within-patient correlation ≈ −0.04).

### 3.6. Temporal Evolution of Mortality

**Figure 5:**
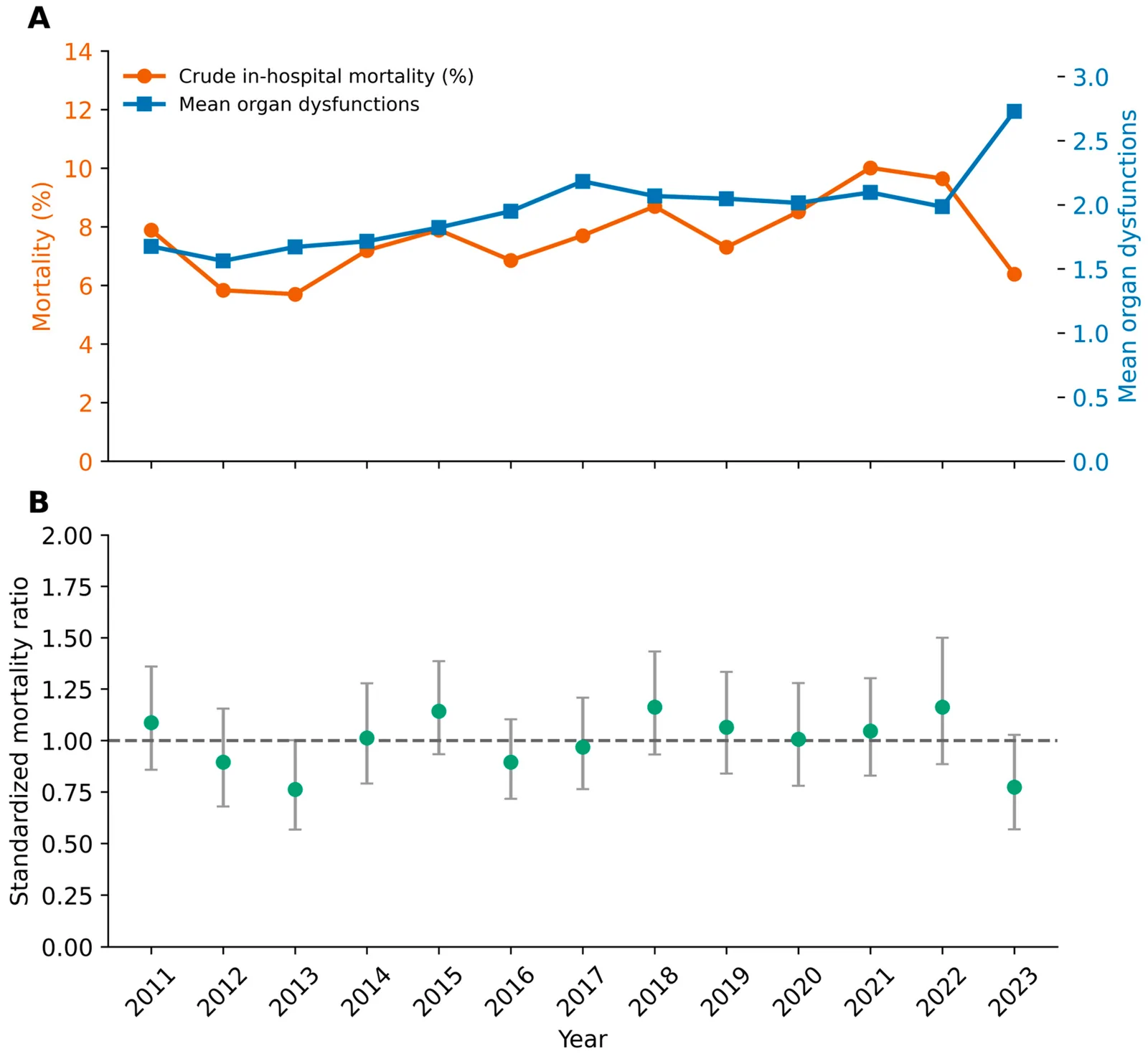
Temporal evolution of mortality and case severity, 2011–2023. (**A**) Annual crude in-hospital mortality (left axis) and mean number of infection-related organ dysfunctions (right axis). (**B**) Standardized mortality ratio (SMR) by year with 95% confidence intervals (CI). The dashed horizontal line indicates the expected value (SMR = 1.0). The figure illustrates the temporal relationship between increasing case severity and stable risk-adjusted mortality.

Crude annual in-hospital mortality ranged between 6% and 10% throughout the study period, with a transient increase during the COVID-19 pandemic years (10.0% in 2021 and 9.6% in 2022) followed by a decrease to 6.4% in 2023. Overall, the crude temporal trend was slightly upward (OR 1.026 per year, *p* = 0.002) (Figure 5A).

Over the same period, the severity of patients attended by the MSU increased. The mean number of infection-related organ dysfunctions was 1.67 in 2011 and 2.72 in 2023 (*p* < 0.001), but the increase was not continuous: the annual mean remained close to 2.0 between 2017 and 2022 and rose most markedly in 2023 (Figure 5). The prevalence of septic shock increased from 8% to 13% (*p* < 0.001), whereas patient age remained relatively stable. ICU admission also varied over time, increasing from 28.5% overall to 35–38% during 2020–2021 before declining to 24% in 2023.

After adjustment for age, ICU admission, infectious focus and severity, calendar year was no longer associated with in-hospital mortality (adjusted OR 1.004 per year, 95% CI 0.98–1.02; *p* = 0.70). Analysis using restricted cubic splines showed no evidence of a non-linear temporal association between calendar year and mortality (*p* = 0.60 for non-linearity).

The standardized mortality ratio remained close to 1.0 throughout the study period, with no significant temporal trend (*p* = 0.71) and no increase during the pandemic years (Figure 5B).

## 4. Discussion

Over thirteen years, a single hospital-wide MSU attended to more than 10,000 patients through more than 50,000 consultations, identified sepsis through multiple referral pathways, actively influenced antimicrobial management in most events, and provided longitudinal follow-up well beyond the initial assessment. During the same period, the severity of patients managed by the unit increased substantially, while risk-adjusted in-hospital mortality remained stable. Taken together, these findings describe the long-term functioning of a mature multidisciplinary sepsis program operating continuously across an entire hospital.

Most organized responses to sepsis focus on a specific stage of the care pathway. Emergency-department sepsis codes aim to accelerate recognition and treatment, screening systems seek to improve early detection, and antimicrobial stewardship programs focus on optimizing therapy [5,6]. Current Surviving Sepsis Campaign guidelines emphasize the importance of performance-improvement programs and code-sepsis systems as key organizational components of sepsis care [7]. The present study suggests that these elements can be integrated within a single operational structure. What distinguishes the model described here is not a specific intervention or technology, but the combination of integration, continuity and permanence. A single multidisciplinary team was responsible for patient identification, bedside evaluation, therapeutic optimization and longitudinal reassessment across the entire hospital over more than a decade.

Two organizational features deserve particular attention. First, patient identification relied on multiple complementary referral pathways rather than a single trigger [13]. Throughout the study period, referrals originated from sepsis-code activations, microbiology alerts, proactive detection systems and direct requests from physicians and pharmacists. Second, patient management extended well beyond the initial consultation. Nearly two-thirds of events received follow-up assessments, with a median of three follow-up visits per event, and follow-up consultations accounted for more than two-thirds of all recorded contacts. Most sepsis pathways are organized around recognition and initial treatment [8,20,21,22,23,24]; by contrast, the present model incorporated longitudinal reassessment as a routine component of care. Such an approach may facilitate ongoing optimization of antimicrobial therapy, reassessment of source control needs and early identification of clinical deterioration throughout the course of infection.

A further notable finding was the extent of antimicrobial stewardship activity. More than half of all events generated therapeutic recommendations, and antimicrobial modification was recommended in 57% of cases. Moreover, nearly 90% of recommended antimicrobial changes were subsequently implemented. These findings suggest that the unit functioned not only as a sepsis detection and response mechanism but also as an effective structure for translating multidisciplinary expertise into routine therapeutic decision-making. The high implementation rate observed may reflect the close integration of the unit within daily clinical practice and its transversal role across multiple hospital departments [25].

The proactive component of case detection evolved substantially during the study period. Initial rule-based electronic alerts were progressively replaced (in 2019) by a more sophisticated machine-learning model, which was subsequently implemented hospital-wide [13,15,16]. Despite these technological changes, the underlying organizational model remained stable. The progressive increase in referrals generated through proactive detection, together with the relative reduction in reliance on manually activated sepsis codes, illustrates how a multidisciplinary sepsis program can incorporate successive generations of detection technologies without requiring major structural changes. The performance of these detection systems has been reported elsewhere [15]; the present study places them within the broader context of long-term program development and clinical activity.

The most important finding of the study was the dissociation between increasing patient severity and stable risk-adjusted mortality over time [26]. Crude mortality increased slightly during the study period, particularly during the COVID-19 pandemic years; however, this increase was accompanied by a marked rise in severity, as reflected by the increasing burden of organ dysfunction and higher prevalence of septic shock. After adjustment for age, ICU admission, infectious focus and severity, no temporal association between calendar year and mortality remained [27,28]. The standardized mortality ratio, which compares observed deaths with those predicted by our own severity-adjusted model, remained close to unity throughout the study period. Because this ratio is internally calibrated, it reflects the temporal consistency of risk-adjusted mortality rather than performance against an external benchmark, and it should not be read as conclusive evidence of the effectiveness of the unit. These findings are consistent with a mature program maintaining stable outcomes despite progressively increasing patient acuity [26]. These findings complement previous work describing the decline in mortality during the early implementation phase of the institutional sepsis program [9]. That study covered a different period and analyzed sepsis-code activations, whereas the present study analyzes MSU-treated events; the two are therefore complementary rather than a single continuous series, and the sequence of an early decline followed by prolonged stability should be read as consistent but indirect evidence rather than a within-study trajectory. In the context of a growing global sepsis burden [2], the maintenance of stable risk-adjusted outcomes over more than a decade may be considered a meaningful organizational achievement.

The factors independently associated with mortality were consistent with established sepsis pathophysiology. Advanced age, ICU admission and increasing numbers of organ dysfunctions were all strongly associated with death, with organ dysfunction burden displaying a clear dose–response relationship [29]. Infectious focus also influenced prognosis, with respiratory, skin and soft-tissue, and unidentified-focus infections associated with substantially higher mortality than urinary infections [30]. The higher mortality of events without an identified focus (adjusted OR 2.55) deserves comment. This group is likely heterogeneous: some cases may reflect delayed identification or control of the source, others an occult bacteremia or a source that was never localized, and others non-infectious conditions initially managed as sepsis, which the unit recorded separately once recognized as non-infectious. Documented bacteremia in the no-focus group was lower than in the cohort overall (9.1% vs. 13.1%), which argues against occult bacteremia as the main driver and is more consistent with a mixed etiology. Septic shock was not independently associated with mortality after adjustment for organ dysfunction burden, likely reflecting the close relationship between these measures of disease severity.

Several limitations should also be considered. The study was observational and conducted in a single center; therefore, the findings demonstrate feasibility and association rather than causation, and external validity requires confirmation in other settings. The absence of a contemporaneous control group precludes direct attribution of observed outcomes to the multidisciplinary sepsis unit itself. Severity data were available for only a subset of events, restricting severity-adjusted analyses to those records. In-hospital mortality was recorded at the admission level and assigned to the terminal event of each admission, a strategy that may not fully separate the outcomes of closely spaced events. Therefore, mortality cannot always be attributed to a specific event among closely spaced events, and the temporal mortality trends are best interpreted at the level of the program rather than the individual event. Successive generations of proactive detection systems could not be distinguished within the registry, and the hospital-wide deployment of machine-learning detection coincided closely with the COVID-19 pandemic, preventing an unconfounded assessment of its impact [15,16]. The standardized mortality ratio was internally calibrated and therefore reflects temporal stability rather than performance against an external benchmark. Finally, the operational sepsis definition was intentionally maintained throughout the study period to ensure longitudinal comparability and therefore does not fully align with contemporary Sepsis-3 definitions. Because separate events required index consultations at least 48 h apart, a clinical deterioration reassessed as a new index consultation after this interval could occasionally be counted as a separate event when it in fact represented the same complicated process, such as treatment failure or an uncontrolled source. This risk is mitigated because clinical deterioration is recorded as a specific field within follow-up consultations, so a worsening course is usually captured as a follow-up of the same event rather than as a new index consultation; nonetheless, a modest over-counting of events relative to distinct infectious episodes cannot be fully excluded. Species-level identification and antimicrobial-resistance phenotypes are held in a separate microbiology database that was outside the data-use scope of this study; we could therefore report the frequency of bacteraemia and of positive cultures but not the distribution of causative pathogens or their resistance patterns. This limits inference about the microbiological appropriateness of empirical therapy, although it does not affect the stewardship-activity measures reported here.

Several strengths support the validity of the findings. The study was based on a prospectively maintained registry spanning thirteen consecutive years of uninterrupted operation and included more than 15,000 events and over 50,000 consultations. The registry captured not only patient characteristics and outcomes but also detailed information regarding referral pathways, clinical interventions and longitudinal follow-up activity. Severity was assigned at the event level through linkage with the institutional sepsis-protocol database, and the main mortality analyses remained robust after accounting for repeated events within individual patients. Few studies have described the long-term activity of a hospital-wide multidisciplinary sepsis program at this scale and level of detail.

Taken together, these findings support the feasibility and long-term sustainability of a permanent, integrated, hospital-wide multidisciplinary sepsis unit operating at scale. The results suggest that the durable elements of successful sepsis programs may depend less on any individual detection technology and more on the organizational infrastructure that integrates early recognition, multidisciplinary clinical response and longitudinal patient follow-up. For hospitals developing or expanding multidisciplinary sepsis pathways, the combination of multi-channel detection, a transversal specialist team and sustained follow-up may represent key organizational components. Whether such an integrated model improves outcomes beyond those achieved by its individual components will require prospective comparative studies and multicenter evaluation. The present findings suggest that the multidisciplinary sepsis unit functions less as a single clinical intervention and more as a hospital-wide organizational layer integrating multiple sepsis-related processes.

## 5. Conclusions

Over thirteen years, this hospital-wide MSU delivered large-scale, longitudinal care to an increasingly severe population while maintaining stable risk-adjusted in-hospital mortality, including throughout the COVID-19 pandemic. Beyond patient outcomes, the study demonstrates the long-term feasibility of an organizational model that integrates multichannel detection, multidisciplinary clinical decision-making and sustained follow-up within a single hospital-wide structure. These findings suggest that durable sepsis programs may depend as much on organizational integration as on the specific technologies used for case detection.

## Figures and Tables

**Table 1:** Baseline characteristics and clinical profile of the cohort.

Cohort Characteristics	Value
Patients, *n*	10,874
Sepsis events, *n*	15,723
Consultations, *n*	50,925
Index	15,723 (30.9%)
Follow-up	35,202 (69.1%)
Age, years, median [IQR]	67 (55–77)
Age, years, mean ± SD	64.8 ± 16.4
Male sex, *n* (%)	6488 (59.3)
**Follow-up activity *(n = 15,723)***	
Events with ≥1 follow-up, %	61.8
Follow-up visits per event, median (IQR)	3 (1–5)
≥2 consultations on the same day, *n* (% of events)	551 (3.5)
ICU admission, *n* (%)	4481 (28.5)
**Severity *(events linked to protocol record, n = 8035)***	
Organ dysfunctions, median (IQR)	2 (1–4)
Septic shock, *n* (%)	852 (10.6)
Activated protocol with confirmed sepsis by MSU, *n* (%)	8007 (99.7)
**Infectious focus, % *(n = 15,723)***	
Respiratory	27.6
Abdominal	26.4
Urinary	15.6
No focus clinical identified	6.7
Catheter-related/primary bloodstream	6.2
Skin and soft tissue	5.7
Central nervous system	0.5
Non-infectious	1.0
Other	3.4
Not specified (missing registry)	7.1
**Referring service, %**	
Intensive care unit	22.2
Emergency department	18.3
Surgery	16.7
Oncology	13.0
Gastroenterology	7.3
Urology	5.4
Pulmonology	4.5
Internal medicine	3.3
Gynecology	3.1
Post-anesthesia care	2.1
Other	3.6
**In-hospital mortality, % *(n = 15,723/n = 8007)***	
All events	7.6
Protocol-confirmed sepsis	11.8

**Table 2:** Activity, referral pathways and antimicrobial stewardship interventions of the multidisciplinary sepsis unit.

Interventions	n (%)
Antimicrobial stewardship activity	
Events with ≥1 recommendation	9240 (58.8)
Antibiotic change	8995 (57.2)
Recommendation acceptance	89.8% *
Type of intervention	
Spectrum reduction	4105 (26.1)
Treatment discontinuation	2448 (15.6)
Switch to oral therapy	2309 (14.7)
De-escalation	2096 (13.3)
Escalation	1897 (12.1)
Correction of inadequate empirical therapy	1612 (10.3)
Treatment initiation	1110 (7.1)
Dose adjustment	306 (1.9)
Operational activity	
Time spent on index consultation, min, median (IQR)	40 (20–60)

Values are n (% of 15,723 sepsis events) unless otherwise stated. * Recommendation acceptance denotes a suggested antibiotic change recorded in the same or the next consultation of the event (15,045/16,758 suggestions, 89.8%). IQR, interquartile range.

**Table 3:** Crude in-hospital mortality by infectious focus (n = 15,723).

Infectious Focus	Events, n	Deaths, n	Mortality, %
Respiratory	4333	459	10.6
Abdominal	4146	256	6.2
Urinary (reference)	2451	93	3.8
No focus identified	1046	126	12.0
Catheter-related/primary bloodstream	982	78	7.9
Skin and soft tissue	894	40	4.5
Central nervous system	78	7	9.0
Other	528	18	3.4
Not specified	1115	103	9.2

Deaths were assigned to the terminal event of each admission, using the same criterion as the overall in-hospital mortality (7.6%). Within catheter-related/primary bloodstream infections, a small endovascular/endocarditis subgroup (n = 38) had the highest crude mortality (23.7%). Urinary infection was the reference category in the multivariable model.

**Table 4:** Independent predictors of in-hospital mortality (multivariable logistic regression, n = 8035).

Predictor	Adjusted OR (95% CI)	*p*
Age, per 10 years	1.34 (1.27–1.42)	<0.001
ICU admission	2.65 (2.25–3.13)	<0.001
Organ dysfunction, per +1	1.37 (1.29–1.46)	<0.001
Septic shock	0.93 (0.74–1.18)	0.57
Focus: respiratory (vs urinary)	1.80 (1.37–2.37)	<0.001
Focus: abdominal	1.18 (0.89–1.57)	0.26
Focus: catheter/primary bloodstream	1.47 (0.95–2.27)	0.084
Focus: skin and soft tissue	2.12 (1.29–3.48)	0.003
Focus: central nervous system	1.01 (0.30–3.42)	0.98
Focus: no focus identified	2.55 (1.82–3.55)	<0.001
Focus: other	1.76 (1.26–2.45)	<0.001

C-statistic 0.75. Reference category for infectious focus, urinary. CI, confidence interval; ICU, intensive care unit; OR, odds ratio. Model based on the subset of events linked to a protocol record (n = 8035).

## Data Availability

The data that support the findings of this study are available upon reasonable request to the first or corresponding author.

## Abbreviations

The following abbreviations are used in this manuscript:

CI	Confidence interval
COVID-19	Coronavirus disease 2019
ICU	Intensive care unit
IQR	Interquartile range
MSU	Multidisciplinary Sepsis Unit
OR	Odds ratio
PIMIS	Computerized Multidisciplinary and Integral Sepsis Protocol
SD	Standard deviation
SIRS	Systemic inflammatory response syndrome
SMR	Standardized mortality ratio
STROBE	Strengthening the Reporting of Observational Studies in Epidemiology
